# Time to develop severe acute malnutrition and its predictors among children living with HIV in the era of test and treat strategies at South Gondar hospitals, northwest, Ethiopia, 2021: a multicentre retrospective cohort study

**DOI:** 10.1186/s12887-021-03078-0

**Published:** 2022-01-14

**Authors:** Ermias Sisay Chanie, Getasew Legas, Shimeles Biru Zewude, Maru Mekie, Dagne Addisu Sewyew, Enyew Dagnew Yehuala, Abenezer Melkie, Minale Bezie Ambie, Mengesha Assefa, Fitalew Tadele Admasu, Getachew Yideg Yitbarek, Sintayehu Asnakew, Mekuant Mersha, Dejen Getaneh Feleke

**Affiliations:** 1 Department of Pediatrics and Child Health Nursing, College of Health Sciences, Debre Tabor University, Debre Tabor, Ethiopia; 2 Department of Psychiatry, school of medicine, College of Health Sciences, Debre Tabor University, Debre Tabor, Ethiopia; 3 Department of Midwifery, College of Health Sciences, Debre Tabor University, Debre Tabor, Ethiopia; 4 Department of Public health, College of Health Sciences, Debre Tabor University, Debre Tabor, Ethiopia; 5 Department of Biomedical Science, College of Health Sciences, Debre Tabor University, Debre Tabor, Ethiopia

**Keywords:** Time to develop severe acute malnutrition, Children living HIV, Ethiopia

## Abstract

**Background:**

Although severe acute malnutrition is a major public issue among HIV infected children, there is no prior evidence in Ethiopia. Hence, this study aims to assess the time to develop severe acute malnutrition and its predictors among children living with human immunodeficiency virus in Ethiopia, 2012.

**Methods:**

An institution based retrospective cohort study was conducted in South Gondar hospitals among 363 HIV infected children from February 10, 2014, to January 7, 2021. Epi-data version 3.1 was used to enter data, which was then exported to STATA version 14 for analysis. Besides, WHO (World Health Organization) Anthro Plus software was used to assess the nutritional status of the children. A standardized data extraction tool was used to collect the data. The Kaplan Meier survival curve was used to estimate the median survival time. The Cox-proportional hazard model assumption was checked via the Schoenfeld residual ph test and a stph plot. Bivariable and multivariable Cox proportional hazard models were employed at 95% confidence intervals (CI). A variable having a *p*-value < 0.05 was considered a statistically significant predictor of severe acute malnutrition.

**Results:**

A total of 363 children living with HIV, 97 (26.72%) developed severe acute malnutrition during the follow-up period. The overall incidence rate was 5.4 (95% CI: 4.7–5.9) person per year with a total of 21, 492 months or 1791 years of observation. Moreover, the median survival time was 126 months. Treatment failure [AHR =3.4 (95% CI: 2.05–5.75)], CD4 count below threshold [AHR =2.5 (95% CI: 1.64–3.95)], and WHO stage III & IV [AHR =2.9 (95% CI: 1.74–4.73)] were all significant predictors of severe acute malnutrition.

**Conclusion:**

The time to develop severe acute malnutrition was found to be very low. Treatment failure, CD4 count below threshold, and WHO stage III were all significant predictors of severe acute malnutrition. Hence, emphasizing those predictor variables is essential for preventing and controlling the occurrence of severe acute malnutrition among HIV infected children.

## Background

In 2019, 1.7 million children worldwide are infected with the human immunodeficiency virus (HIV) [1]; in the same year, 44,229 children in Ethiopia were infected with HIV, and 2055 died as a result of AIDS (Acquired immunodeficiency syndrome) [2].

HIV infection and malnutrition are often closely interlinked and act synergistically [3, 4]. Malnutrition increases viral replication and accelerates the progression of HIV disease [5, 6]. Likewise, the effect of HIV on nutrition includes nutrient malabsorption and complex metabolic alterations [3, 7]. As a result, a vicious cycle is formed [8, 9].

The incidence of stunting is declining too slowly, while wasting still has a great impact on many young children worldwide [10]. In 2017, 13.8 million children were wasted, of whom 4 million were severely wasted [11] and most of all were in sub-Saharan countries [12]. Each year, over one million children die and develop severe acute malnutrition (SAM), and it is more common in HIV-positive children [8, 13, 14]. Moreover, SAM among HIV-infected children is one of the leading causes of morbidity and mortality in the world, and the problems are worsening more in sub-Saharan countries [13, 15, 16]**,** and Ethiopia is one of those countries to share this burden [13, 17].

Children are more vulnerable to severe acute malnutrition than adults with HIV, hence rapid viral replication and a higher rate of CD4 cell destruction because of immunity that has not developed well [15]. The risk of death in HIV-infected children is three times higher than in non-HIV-infected children [7, 18]. Since HIV increases the risk of loss of appetite, worsening illness, and deteriorating nutritional status [19, 20]. As a result of HIV prevalence and resource constraints, caring for severely acute malnourished children in Sub-Saharan Africa, such as Ethiopia, is difficult [6, 12].

SAM among HIV-infected children remains widespread and a serious public health problem in Ethiopia since it is interlinked with different contributing factors [13, 21, 22]**.**

Although SAM is a leading cause of hospitalization and mortality in Ethiopia among HIV-infected children, there is no prior evidence on when to develop SAM and its predictors among HIV-infected children. Hence, this study aims to assess the time to develop severe acute malnutrition and its predictors among HIV-infected children at South Gondar hospital, Ethiopia. Additionally, the finding hopes that it will provide important information to be understood by all stakeholders, reducing the burden.

## Methods

### Study area, design and study participants

A retrospective follow-up study was conducted from February 10, 2014, to January 7, 2021, at South Gondar hospitals. The South Gondar Zone is one of the zonal states of the Amhara region. Based on the 2007 census conducted by the Ethiopian Central Statistical Agency, this zone has a total population of 2,051,738, of whom 1,041,061 are males, while 1,010,677 are females. South Gondar hospitals provide different services for the South Gondar Zone population, such as services in the MCH unit, genecology and obstetrics unit, laboratory unit, minor surgery, inpatient unit, and outpatient unit, major surgery, ophthalmic unit, pharmacy unit, and ART. Besides, the South Gondar public hospitals have 612 health professionals. During the study period, there were 1027 children (age 15 years) on ART, according to the South Gondar administrator report.

All HIV infected children from February 10, 2014, to January 7, 2021, at South Gondar hospitals were a study population. All children with HIV after the beginning of test and treat strategies until the end of the study period were eligible for the study. Whereas, children with unrecorded nutritional status were excluded from the study.

### Sample size determination and sampling procedure

The sample size was calculated by using the Cox proportional hazards model via Stata 14.0 software using the two-population proportion formula by considering the following assumptions. n = (Zα/2 + Zβ)2 * (p1(1-p1) + p2(1-p2)) / (p1-p2)2, where Zα/2 is the critical value, which is 1.96, Zβ is the critical value with a power of 80%, and the critical value is 0.84. The p1 and p2 are the expected sample proportions of the two groups. Based on the above two-population proportion formula, the survival probability in males as an exposure group (P1 = 0.323) and the survival probability in females as a non-exposure group was (P2 = 0.470) from a previous study [9]. The final sample size *n* = (1.96 + 0.84)^2^ * (0.323 (1–0.323) + 0.470 (1–0.470)) / (0.323–0.470)^2^ after adding 10% incompleteness data was 363. There are a total of 05 hospitals that provide Paediatric ART in the South Gondar Zone. Of these, one compressive specialized hospital and two primary hospitals were selected randomly. Namely, Debre Tabor compressive specialized hospital, Nefas Mewucha primary hospital, and Mekan Eysus primary hospital.

**Fig. 1:**
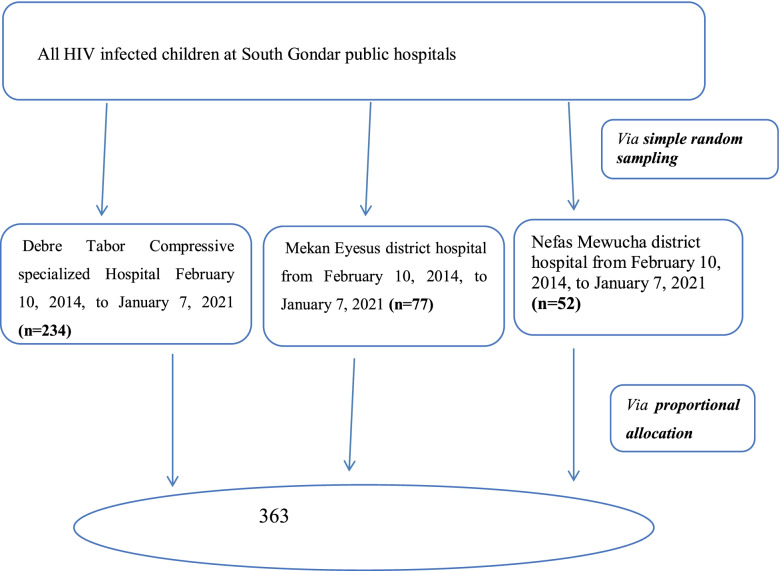
Schematic presentation of study participants’ recruitment and allocation process at south Gondar hospitals, Northwest, Ethiopia, 2021 (n-363)

From a total of 1027 study participants, 719 were in the selected hospitals from February 10, 2014, and January 7, 2021. Then, eligible participants from those selected hospitals were identified at paediatric ART clinics. The investigator assigned the registration numbers from February 10, 2014, and January 7, 2021, in chronological order. Of these, the investigator drew 363 samples by lottery method that fulfilled the inclusion criteria after reviewing the medical charts and ART registration logbook after proportionally allocating them to each hospital (Fig. 1).

### Operational definitions

#### Time to SAM (outcome variable)

The time from admission to the occurrence of the event SAM during the follow-up period.

SAM is defined by the WHO as a weight-for-height z-score of less than − 3, or a mid-upper arm circumference of less than 11.5 cm in children aged 6 months to 5 years [8, 15].

Censored: if the child had lost follow-up or transferred out to another service before developing SAM, or if the child was free from SAM until the end of the follow-up time.

Adherence to ART was classified based on the percentage of drug dosage calculated from the total monthly doses of ART drugs. (Good > 95%, fair 85–94%, and poor < 85%) [23].

Anemia was defined as having a hemoglobin level ≤ 10 mg/dl [24].

CD4 count: CD4 levels below the threshold level were classified based on the child’s age (i.e. infants CD4 1500/mm3, 12–35 months 750/mm3, 36–59 months 350/mm3, and 5 years 200/mm3) [24].

#### Data collection procedures and quality control

Data were collected from children’s follow up by using a standard checklist that was adopted from the ART follow-up form. The data extraction also contained socio-demographic and clinical related characteristics of the children or caregivers. A pre-test was done on 10% of the sample size in Mekane Eyesus primary hospital, which is 52 km away from Debre Tabor Town. Tanning was given for four data collectors and two supervisors. Indeed, the data collectors had been working in ART clinics and knew the format. The completeness of the retrieved data was checked by the supervisor after the randomly selected medical records on a daily basis.

#### Data processing and analysis

For this study, Epi-data version 3.1 Epi-data version 3.1 was used to enter data, which was then exported to STATA version 14 for analysis. Moreover, WHO Anthro Plus software was used to assess the nutritional status of the children. The median, tables, and figures were used to describe and visualize the data. The incidence rate of SAM was calculated using the total number of people per year (PPY) as the denominator in the follow-up period. The total time that participants contributed to the study was thus incorporated into the analysis. The Kaplan-Meier plot estimates the median SAM free survival time.

**Fig. 2:**
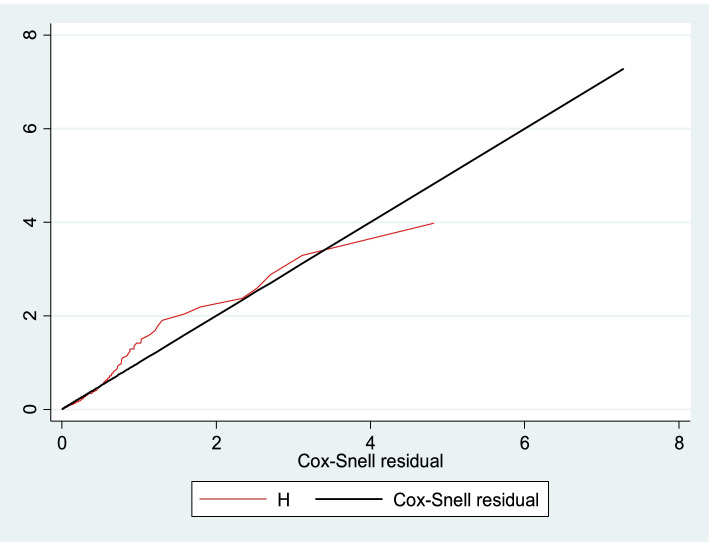
Cox-Snell residual Nelson -Alen cumulative hazard graph among children living with HV at south Gondar hospitals, Northwest, Ethiopia, 2021 (n-363)

The necessary Cox-proportional hazard model assumption was checked using graphical diagnostics based on the scaled Schoenfeld residuals (log-log survival plot) (Fig. 2) and statistical tests (global test: 39.05). After the Cox-proportional hazard model was checked, both bivariate and multivariable Cox proportional hazard regression analyses at 95% CI were fitted. Then, the variables having a *p*-value < 0.25 in bivariate analysis were computed into the multivariable cox-proportional hazards model. Finally, a variable having a *P*-value < 0.05 in the multivariable cox-proportional hazards model was used to declare a significant association with SAM.

## Results

### Socio-demographic characteristics of children living with HIV

Overall, 363 children living with HIV (Debre Tabor compressive specialized hospital: 234, Nefas Mucha primary hospital: 52, and Mekan Eyesus primary hospital: 77) were included in the study. Of the 363 children living with HIV, 190 (52.34%) and 131 (36.09%) were male and aged between 4 and 10, respectively. The majority of the 290 (79.89%) children living with HIV were in urban residences. Besides, a large proportion of 238 (67.23%), 249 (68.6%), and 281 (77.41%) of the child caregivers were married, both alive, and living with HIV, respectively. Almost half of the children (47.9%) were aware of their HIV status. Likewise, approximately 203 (55.92%) of the children’s caregivers were housewives (Table 1).

**Table 1:** Socio-demographic characteristics of children living with HIV at South Gondar hospitals, Ethiopia, 2021(*n* = 363)

Exposure variable	Responses	Frequency	Percent
Age of the child (years)	≤3	95	26.17
	> 4–10	131	36.09
	> 10	137	37.74
Sex	Male	190	52.34
	Female	173	47.66
Residence	Rural	73	20.11
	Urban	290	79.89
Caregiver’s Marital status	Married	238	67.23
	Widowed	106	29.94
	Divorced	10	2.82
Caregiver’s status	Both alive	249	68.6
	Either or Bothe died	114	31.4
Caregiver’s occupational status	Housewife	203	55.92
	Governmental employee	89	24.52
	Merchant	38	10.47
	Non-governmental employee	33	9.09
HIV status of the Caregiver’s	Positive	281	77.41
	Negative	38	10.47
	Not Known	44	12.12
Disclosure status	Yes	174	47.9
	No	189	52.1

### Clinical characteristics of children living with HIV

Of the total of children living with HIV, 49 (13.50%), 61 (16.80%), 81 (22.31%), 40 (11.02%), and 39 (10.74%) had HGB (Hemoglobin) levels of 10 mg/dl, CD4 counts or % below the threshold, WHO stages III & IV, TB (Tuberculosis), and treatment failure in the follow-up period. Additionally, 202 (63.325) children living with HIV had OI (opportunistic infection) in the study period. ART was started on nearly half of the 188 children (51.8%) who were alive after 49 months. Of the total 363 children living with HIV, 300 (82.64%) were taking CPT (Cotrimoxazole prophylactic therapy), while 156 (42.98%) were taking IPT (Isoniazid prophylactic therapy). Besides, 327 (90.08%) and 280 (77.13%) of children living with HIV were initial non-PI (protease inhibitor) based and had a good level of adherence to ART (Table 2).

**Table 2:** Clinical and immunological status characteristics of children living with HIV South Gondar hospitals, Ethiopia, 2021(*n* = 363)

HGB level	< 10 mg/dl	49	13.50
	> = 10 mg/dl	314	86.50
CD4 counts or %	Below threshold	61	16.80
	Above threshold	302	83.20
WHO stages	Stage I&II	282	77.69
	Stage III&IV	81	22.31
Hx of OIs other than TB (*n* = 319)	Yes	117	36.68
	No	202	63.32
Regimen at baseline	PI-based	36	9.92
	Non-PI based	327	90.08
Treatment failure	Yes	39	10.74
	No	324	89.26
Cotrimoxazole preventive therapy (CPT)	Yes	300	82.64
	No	63	17.36
Isoniazid preventive therapy (IPT)	Yes	156	42.98
	No	207	57.02
ART adherence	Good	280	77.13
	Poor/Fair	83	22.87
TB status	Yes	40	11.02
	No	323	88.98
Duration On ART	< 49 months	188	51.8
	≥49 months	175	48.2

### The time to exposure for severe acute malnutrition in HIV-infected children

**Fig. 3:**
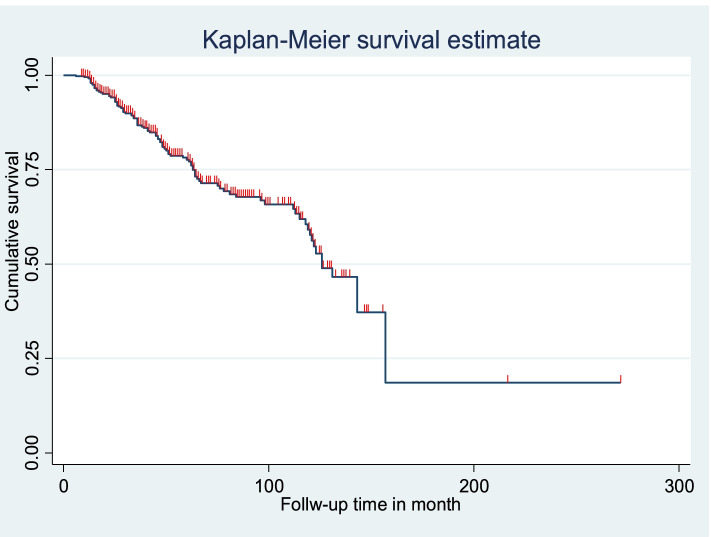
Kaplan-Meier of survival curve among children living with HV at south Gondar hospitals, Northwest, Ethiopia, 2021 (n-363)

From a total of 363 children living with HIV, 97 (26.72%) developed SAM during the follow-up period. The incidence rate of SAM among children living with HIV was 5.4 (95%CI: 4.7–5.9) PPY. The study participants were followed for a total of 21, 492 months, or 1791 at different points of time. The median survival time was 126 months (Fig. 3).

### Predictors of severe acute malnutrition among children living with HIV

**Fig. 4:**
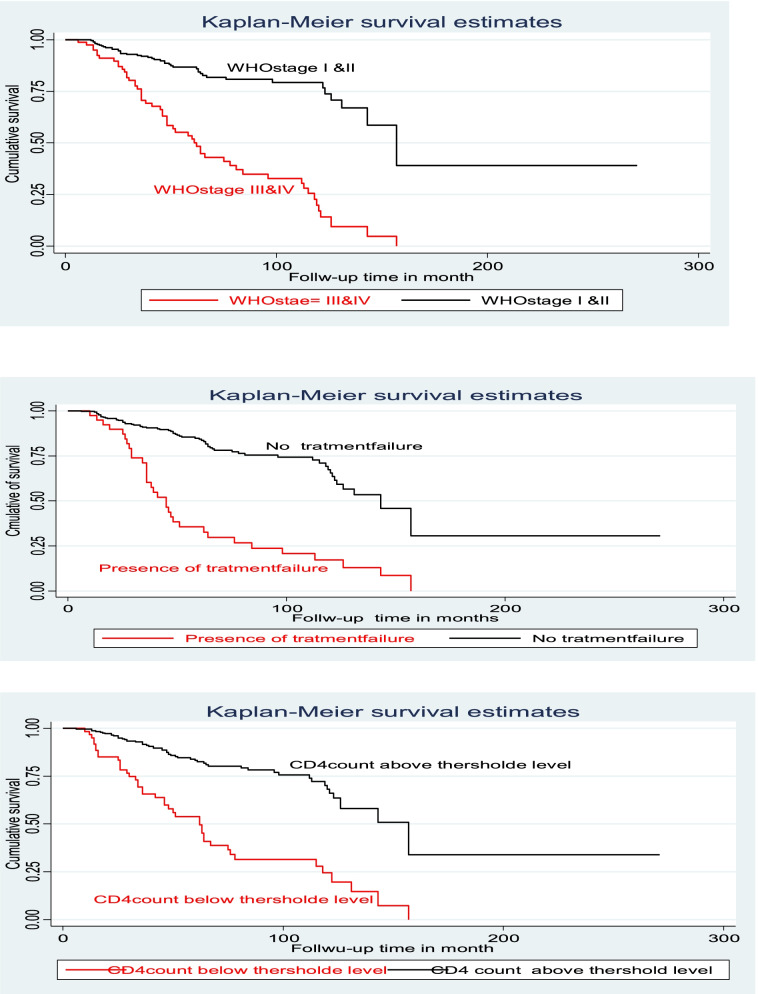
Kaplan-Meier of survival curve of among children living with HV by treatment failure, CD4 count, and WHO stage at south Gondar hospitals, Northwest, Ethiopia, 2021 (n-363)

Bi-variable and multivariate cox proportional hazard models were computed to determine the relationship between each independent factor and the outcome variable. In bi-variable analysis, the variable had a *P*-value less than or equal to 0.25 and was entered into for multivariate cox proportional hazard after checking global and detail tests for the model fitness and for all predictor variables, respectively. In the multivariate cox proportional hazard model, treatment failure, WHO stages III & IV, and CD4 counts below threshold level were significant predictors for the occurrence of SAM among children living with HIV. The risk of SAM among children with treatment failure was 3.4 times higher than that of children without treatment failure [AHR: 3.4 (95% CI: 2.05, 5.75)]. The risk of SAM among children with CD4 counts below the threshold level was 2.5 times higher than that of children with CD4 counts above the threshold [AHR: 2.5 (95% CI: 1.64, 3.95)]. Moreover, the risk of SAM among children with WHO stage III & IV is 2.9 times higher than in children with WHO stage III & IV [AHR: 2.9 (95% CI: 1.74, 4.73)] In the follow-up period (Table 3 &Fig. 4).

**Table 3:** Bivariable and multivariable Cox-regression of predictor variable among children living with HIV at South Gondar hospitals, Ethiopia, 2021(*n* = 363)

Exposure variable		SAM NO Yes		CHR (95% CI)	AHR (95% CI)	*P*-Value
Age of the child (years)	≤3	71	24	1.0 (0.63–1.78)	–	
	> 4–10	96	35	1.0 (0.64–1.60)		
	> 10	99	38	Ref		
Sex	Male	144	46	0.7 (0.47–1.05)	0.9 (0.56–1.32)	0.487
	Female	122	51	Ref	Ref	
Residence	Rural	51	22	1.2 (0.79–1.78)	–	
	Urban	215	75	Ref		
Caregiver’s Marital status	Married	175	63	1.2 (0.37–3.78)	–	
	Widowed	79	27	1.1 (0.34–3.76)	–	
	Divorced	7	3	Ref		
Caregiver’s status	Both alive	186	63	Ref		
	Either or Bothe died	80	34	1.1 (0.71–1.65)	–	
Caregiver’s occupational status	Housewife	145	58	0.8 (0.35–1.70)	–	
	Governmental employee	66	23	0.7 (0.30–1.67)	–	
	Merchant	29	9	0.8 (0.28–2.07)	–	
	Non-governmental employee	26	7	Ref		
HIV status of the Caregiver’s	Positive	218	63	0.5 (0.29–0.83)	0.6 (0.32–1.01)	0.053
	Negative	23	15	0.9 (0.46–1.84)	0.4 (0.18–1.01)	0.054
	Not Known	25	19	Ref	Ref	
Disclosure status	Yes	135	54	Ref	Ref	
	No	131	43	1.3 (0.86–1.92)	1.6 (0.98–2.47)	0.057
Regimen at baseline	PI-based	12	24	3.0 (1.90–4.82)	0.9 (0.50–1.61)	0.715
	Non-PI based	254	73	Ref	Ref	
Treatment failure	Yes	6	33	**4.3 (2.81–6.57)**	**3.4 (2.05–5.75)**	**0.000**
	No	260	64	Ref	Ref	
Cotrimoxazole preventive therapy	Yes	24	39	Ref	Ref	
	No	242	58	3.5 (2.30–5.19)	1.6 (0.96–2.65)	0.070
CD4 counts or %	Below threshold	19	42	**3.9 (2.62–5.85)**	**2.5 (1.64–3.95)**	**0.000**
	Above threshold	247	55	Ref	Ref	
Isoniazid preventive therapy (IPT)	Yes	125	31	Ref	Ref	
	No	141	66	1.3 (0.85–2.02)	0.8 (0.49–1.26)	0.327
ART adherence	Good	203	77	Ref		
	Poor/Fair	63	20	0.8 (0.49–1.32)		
TB status	Yes	12	28	3.7 (2.37–5.75)	1.2 (0.68–2.27)	0.471
	No	254	69	Ref	Ref	
Hx of OIs other than TB (n = 319)	Yes	85	32	1.1 (0.68–1.64)	–	
	No	149	53	Ref		
Hemoglobin level	< 10 mg/dl	19	30	2.4 (1.55–3.71)	1.6 (0.75–1.95)	0.433
	> = 10 mg/dl	247	67	Ref	Ref	
WHO stages	Stage I&II	239	43	Ref	Ref	
	Stage III&IV	27	54	**4.8 (3.18–7.11)**	**2.9 (1.74–4.73)**	**0.000**
Duration On ART	< 49 months	131	57	0.9 (0.6–1.4)	–	
	≥49 months	135	40	Ref		

*Significant at < 0.05; ** Significant at < 0.01; *CHR* Crude hazard ratio, *AHR* Adjusted hazard ratio, *Ref* Reference category, *CI* Confidence interval

## Discussion

In this study, the average time to exposure for severe acute malnutrition at South Gondar hospitals was found to be 126 months. Besides, the incidence of severe acute malnutrition was found to be 5.4 (95% CI: 4.7–5.9) PPY with a proportion of 26.7%.

This finding is comparable to 24.65% in East Africa [8], 24.5% in sub-Saharan Africa [25], and 26% in Central and West Africa [12]. However, this finding is higher than the studies conducted in Asia 13.5% [26]**,** 12.9% in Tanzania [27], 13.6% in Nigeria [28], 10.6**%** in Cameron **[**29]**,** and 7.1% in Zimbabwe [30]. On the other hand, the finding was lower than the study conducted in India, it was 38.09% **[**31**]**. These differences could be explained by the level of quality care, which includes the reporting mechanism, the cut-off point for SAM diagnosis, screening, and monitoring ability of the healthcare providers. Moreover, the differences in socio-demographic characteristics, healthcare systems, study population, and study design were also considered for the variation.

The risk of SAM among children with treatment failure was 3.4 times higher than that of children without treatment failure [AHR: 3.4 (95% CI: 2.05, 5.75)]. This study’s findings were consistent with those conducted in Asia and Burkina Faso [26, 32]. This can be explained by the fact that children with treatment failure are at risk for viral load increment and the CD4 cell count continues to drop, which leads to the development of an opportunistic infection like SAM. Besides, antiretroviral therapy can prevent HIV from progressing, especially when a person starts taking it early. However, if the patients have treatment failure, HIV progressively kills the cells like CD4 T cells that help protect the body from infection, leading to complications [33–35].

The risk of SAM among children with CD4 counts below the threshold level was 2.5 times higher than that of children with CD4 counts above the threshold [AHR: 2.5 (95% CI: 1.64, 3.95)].

This might be because the patient who has a low CD4 count can be exposed to chronic diarrhea, tuberculosis, opportunistic infections, or anemia, which results in a significant imbalance between nutritional demand and individual intake, usually both quantitative (number of kilocalories/day) and qualitative (vitamins and minerals, etc.) deficiencies [36, 37].

Moreover, the risk of SAM among children with WHO stage III & IV is 2.9 times higher than in children with WHO stage III & IV [AHR: 2.9 (95% CI: 1.74, 4.73)] This study’s findings were consistent with the studies conducted in Asia and Burkina Faso [38, 39]. This is usually when children are presented with advanced WHO stages, where the immune system is badly damaged, which exposes them to different kinds of complications, including cancer, pneumonia, treatment failure, diarrhea, and anemia. Hence, if the children are affected severely by advanced opportunistic infections, they are unprotected against chronic and acute malnutrition [40, 41].

This finding of this study has an implication for the global rise of severe acute malnutrition, predominantly in resource-limited settings. It adds to an understanding of the links between SAM and the contributing factors that cause its occurrence in children living with HIV. These findings provide an insight into effective ways of improving the quality-of-life of children with HIV in Ethiopia, a country known for its high prevalence of severe acute malnutrition, especially among children living with HIV. Additionally, these findings may help policymakers establish context-specific strategies to reduce severe acute malnutrition, particularly in children with HIV [8, 13, 42**]**.

This study can try to show the burden of SAM plus attempt to establish the cause-effect relationship between SAM and its predictor variable. However, the study’s inherent limitations of retrospective study design were encountered.

## Conclusion

The time to develop severe acute malnutrition was found to be very low. Treatment failure, CD4 count below threshold, and WHO stage III were all significant predictors of severe acute malnutrition. Hence, emphasizing those predictor variables is essential for preventing and controlling the occurrence of severe acute malnutrition among HIV infected children.

## Data Availability

The datasets used and/or analyzed during the current study are available from the corresponding author.

## Abbreviations

AHR: Adjusted hazard ratio
AIDS: Acquired immunodeficiency disease
ART: Antiretroviral therapy
CD4: Cluster of differentiation 4
CI: Confidence interval
CPT: Cotrimoxazole prophylactic therapy
HGB: Hemoglobin
HIV: Human immunodeficiency virus
IPT: Isoniazid prophylactic therapy
TB: Tuberculosis
OI: Opportunistic infection
PI: protease inhibitor
PYO: Person per year observation
WHO: World health organization
